# Beta endorphin in serum and follicular fluid of PCOS- and non-PCOS women

**DOI:** 10.1007/s00404-018-4793-6

**Published:** 2018-05-28

**Authors:** Nikolai Jaschke, Fabian Lunger, Ludwig Wildt, Beata Seeber

**Affiliations:** Department for Gynecological Endocrinology, Medical University of Innsbruck, Anichstrasse 35, 6020 Innsbruck, Austria

**Keywords:** Opioids, Granulosa cells, PCOS, Beta endorphin

## Abstract

**Purpose:**

To compare the concentrations of beta endorphin in serum and follicular fluid (FF) of PCOS- and non-PCOS women. Secondarily, to investigate associations between beta endorphin and other parameters.

**Methods:**

Fifty-nine women undergoing in vitro fertilization (IVF) were included in the study. Sixteen were stratified to the PCOS group using the Rotterdam criteria. The remaining 43 women served as controls. Follicular fluid was collected during oocyte retrieval and peripheral blood sampling was performed on the same day. Beta endorphin concentrations in serum and follicular fluid, serum levels of insulin, glucose, LH, estradiol and progesterone were measured. Additionally, testosterone was measured before starting the stimulation protocol.

**Results:**

There was no difference in beta endorphin levels between PCOS- and non-PCOS women. The concentration of the peptide was higher in serum than in FF, likely due to collection of FF after ovulation induction and corresponding to the early luteal phase. We found a significant correlation between the number of mature Metaphase II (MII) oocytes retrieved and beta endorphin concentration in FF. In women with biochemical hyperandrogenemia, beta endorphin levels in FF correlated with testosterone levels.

**Conclusion:**

Beta Endorphin concentrations in serum and FF do not differ between PCOS- and non PCOS-women undergoing IVF. However, together with sex hormones, beta endorphin might play a key role in oocyte maturation.

## Introduction

The polycystic ovary syndrome (PCOS) is one of the most common endocrinopathies of women in reproductive age. Many hypotheses regarding the pathogenesis of the disorder have been postulated. Decades ago, neuroendocrine abnormalities were thought to play a causative role [1]. 30 years later, Gilling-Smith et al. reported about a primary dysfunction of ovarian steroidhormone synthesis in PCOS women [2].

The role of endogenous opioids, especially the POMC-derivate beta endorphin, in PCOS has been a research topic for some time [3–5], but has not been extensively studied.

All endogenous opioids share the same n-terminal aminoacid pentasequence, the so-called “opioid motif” [6]. Preopiomelanocortin (POMC), preprodynorphine and preproenkephaline are post-translationally processed and function as precursor molecules for the different opioid peptides [7–9]. Three main opioid receptor classes have been described: *µ*, *κ* and *δ* [10]. Although all opioid peptides can bind to each receptor subclass, their affinity for the different receptors vary [11]. While the role of endogenous opioids in the neuroendocrine regulation of the menstrual cycle has been well established [12, 13], very little is known about the effects of these molecules in the periphery. Beta endorphin has been shown to modulate pancreatic insulin and glucagon release [14]. Interestingly, the presence of sex steroids seems to be critical for beta endorphin action in the human pancreas [5].

In 1985, Petraglia et al. first reported that beta endorphin in human follicular fluid showed higher concentrations in the follicular phase of the menstrual cycle and lower concentrations in the luteal phase [15]. In postmenopausal women, no beta endorphin was detectable in follicular fluid (FF). More recently, the expression of opioid receptors on human oocytes, as well as follicular granulosa cells has been confirmed [16, 17]. Together these data indicate a putative role of opioids, especially beta endorphin, in reproductive function of women, both centrally and in the periphery (i.e. in the ovary). Specifically, altered opioid-induced regulation of granulosa cell VEGF expression has been suggested in PCOS- compared to non-PCOS women, leading to increased VEGF secretion in the former [18].

Based on these prior studies, we hypothesized that (1) concentrations of beta endorphin are higher in follicular fluid than in serum, thus indicating a local production from granulosa cells and/or the oocyte and (2) concentrations of beta endorphin in FF und serum differ between PCOS- and non-PCOS women.

## Materials and methods

The study was approved by the ethical committee of the Medical University of Innsbruck, Innsbruck, Austria.

Fifty-nine reproductive age women (21–43 years) who presented for in vitro fertilization (IVF) between 2014 and 2016 were included in the study after obtaining informed consent. We used the Rotterdam Criteria [19] to differentiate between PCOS- and non-PCOS women. Specifically, biochemical hyperandrogenemia was defined as total testosterone > 0.4 µg/l measured by liquid chromatography-mass spectrometry (LC/MS) as established in our center. Oligomenorrhea was defined as a menstrual cycle > 35 days, and amenorrhea as the absence of menstruation for > 3 months. The only exclusion criteria were the presence of endometriosis and age < 18.

The subjects underwent ovarian stimulation in agonist- or antagonist-protocols, as routinely performed in our clinic. In women with oligomenorrhea, stimulation was begun after inducing a withdrawal bleed with progesterone and without the routine use of oral contraceptives. 36 h after ovulation induction using hCG or GnRH agonist, follicles were punctured, oocytes retrieved and FF was collected. The FF from the first follicle punctured from each side was collected separately without flushing. With this method, we aimed to minimize dilution effects. Subsequently, the follicular fluids of these two follicles were pooled, immediately centrifuged and stored at – 80 °C until the day of the assay.

Serum samples for beta endorphin were collected on retrieval day (corresponding to the early luteal phase) and were stored at – 80 °C until analysis. In addition, fasting insulin, glucose, LH, estradiol and progesterone were measured per routine by the central laboratory of the Medical University of Innsbruck. Testosterone was measured before starting the stimulation protocol.

In both the sample types (FF and serum), beta endorphin was measured with a test kit (mdbioproducts, Egg near Zurich, Switzerland) using enzyme-linked immunosorbent (ELISA) technique with an assay sensitivity of 1 ng/ml and no cross-reactivity between beta endorphin and other endogenous opioids.

### Statistical analysis

Baseline characteristics of the two study groups are expressed as mean ± SD or median with interquartile range.

Between-group comparisons of variables were performed using unpaired *t* test and Mann–Whitney *U* test. Paired *t* test was used to compare beta endorphin concentrations in serum and FF. Spearman correlation analysis was used for investigating associations between different variables.

To determine which variables were independent predictive factors for number of retrieved mature MII (Metaphase II) oocytes and beta endorphin levels in FF, respectively, we performed linear regression analysis.

All statistical analyses were performed using SPSS Version 22 (SPSS Inc., Chicago, IL).

*P* values < 0.05 were considered significant.

## Results

Of the 59 women included in our study, 16 were stratified to the PCOS group according to the Rotterdam criteria. The remaining 43 women were assigned to the control group.

Patient characteristics are shown in Table 1. Women in the PCOS group were significantly younger than controls, but no differences in BMI were noted. As expected, women in the PCOS group had higher baseline testosterone values and had higher estradiol and progesterone concentrations on retrieval day than women in the control group, the latter two measurements being proportional to the higher number of follicles and oocytes produced. The two groups did not differ in fasting insulin and glucose measurements.

**Table 1:** Patient characteritics

	Control	PCOS	*p* value
Age	34.7 ± 5.0	29.3 ± 4.0	< 0.000
BMI	23.7 ± 5.1	25.2 ± 6.4	0.374
Insulin (U/l)	7.4 (6.8)	12.0 (12.3)	0.171
Glucose (mg/dl)	84 (14)	89 (22)	0.756
LH (U/l)	0.95 (1.9)	0.8 (3.0)	0.519
Progesterone (µg/l)	5.75 (5.5)	10.65 (12.0)	0.011
Estradiol (ng/l)	1161 (815)	1606 (1244)	0.018
Testosterone (µg/l)	0.21 (0.13)	0.35 (0.31)	0.003
Number of oocytes	8 (8)	21 (15)	< 0.000
Number of MII	7 (8)	18 (13)	< 0.000
BetaEnd_S (ng/ml)	0.64 (0.31)	0.53 (0.36)	0.186
BetaEnd_FF (ng/ml)	0.37 (0.28)	0.46 (0.23)	0.117

Data presented as mean ± SD and median with interquartile range, respectively

*MII* MetaphaseII oocytes, *BetaEnd_S* beta endorphin in serum, *BetaEnd_FF* beta endorphin in follicular fluid

Furthermore, significantly more oocytes and mature oocytes (metaphase II oocytes) were retrieved in the PCOS group.

12 women (75%) in the PCOS group were treated using an antagonist protocol and the remaining 4 (25%) with an agonist protocol. In the control group, 17 (40%) were treated with an agonist-, 26 (60%) with an antagonist-protocol, respectively. There was no difference in distribution among the two protocols between the PCOS-group and the control-group (*p* = 0.365).

There was no difference in the concentration of beta endorphin in serum, nor in FF between the two groups, 0.64 versus 0.53 ng/ml (*p* = 0.186) and 0.37 versus 0.46 ng/ml (*p* = 0.117). Unexpectedly, for all women, we found significantly lower beta endorphin concentrations in FF than in serum (0.45 vs. 0.65 ng/ml, *p* = 0.001).

We further elucidated which potential factors predicted the number of retrieved MII oocytes using linear regression analysis (least square fit) with number of MII oocytes as the dependent variable. Estradiol, progesterone, testosterone, insulin, beta endorphin concentration in FF and age were used as independent variables. The results are shown in Table 2.

**Table 2:** Regression analysis with number of MII oocytes as the dependent variable

	E2	P	T	Insulin	Beta endorphin FF	Age
MII oocytes	*r*^2^ = 0.097	*r*^2^ = 0.331	*r*^2^ = 0.196	*r*^2^ = 0.079	*r*^2^ = 0.115	*r*^2^ = 0.395
	*B *= 0.002	*B *= 0.622	*B* = 25.05	*B *= 0.312	*B* = 12.26	*B *= − 0.923
	*p* = 0.016	*p* ≤ 0.000	*p* ≤ 0.000	*p* = 0.034	*p* = 0.044	*p* ≤ 0.000

In addition to age, beta endorphin and testosterone were each found to be independent predictors with the highest regression coefficients of 12.26 and 25.05, respectively. This indicates that for each increase of testosterone and beta endorphin by one unit (1 µg/l or 1 ng/ml), the number of retrieved MII oocytes increases by the above noted coefficient.

**Fig. 1:**
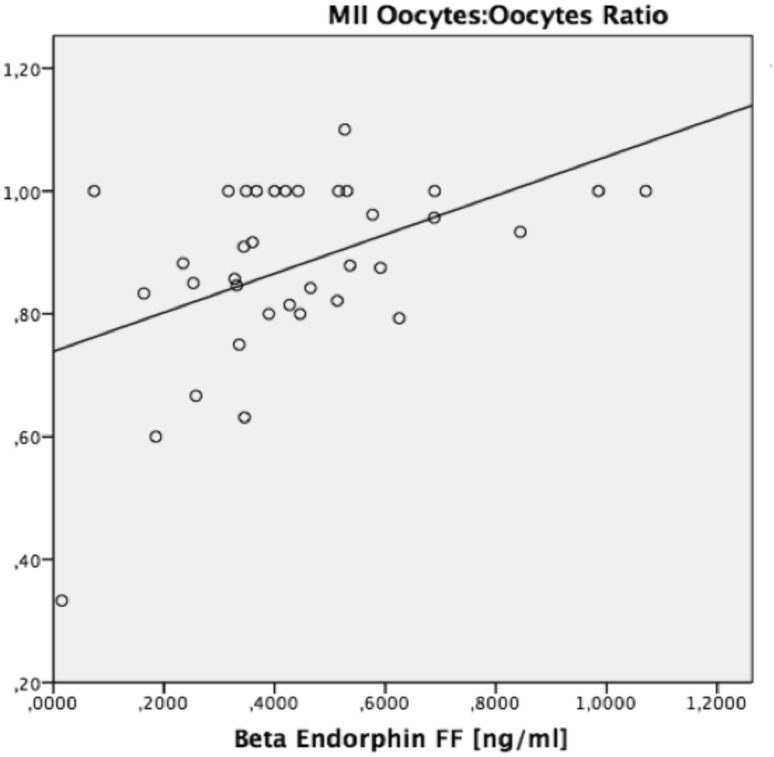
Regression analysis: *r*^2^ = 0.228, *p* = 0.003

With an increasing number of total oocytes isolated, the probability for a higher number of retrieved MII oocytes also increases. Thus, we calculated a ratio of MII oocytes:number of oocytes. This ratio also correlated significantly with beta endorphin content in FF (*r*^2^ = 0.228, *p* = 0.003) (Fig. 1).

We found no correlation between beta endorphin concentration in serum and FF (*p* = 0.353).

Similarly, we performed regression analysis to evaluate which independent variables (age, insulin, LH, progesterone, estradiol, testosterone) predicted beta endorphin levels in FF. None of the variables measured correlated with beta endorphin concentration in FF or serum.

**Fig. 2:**
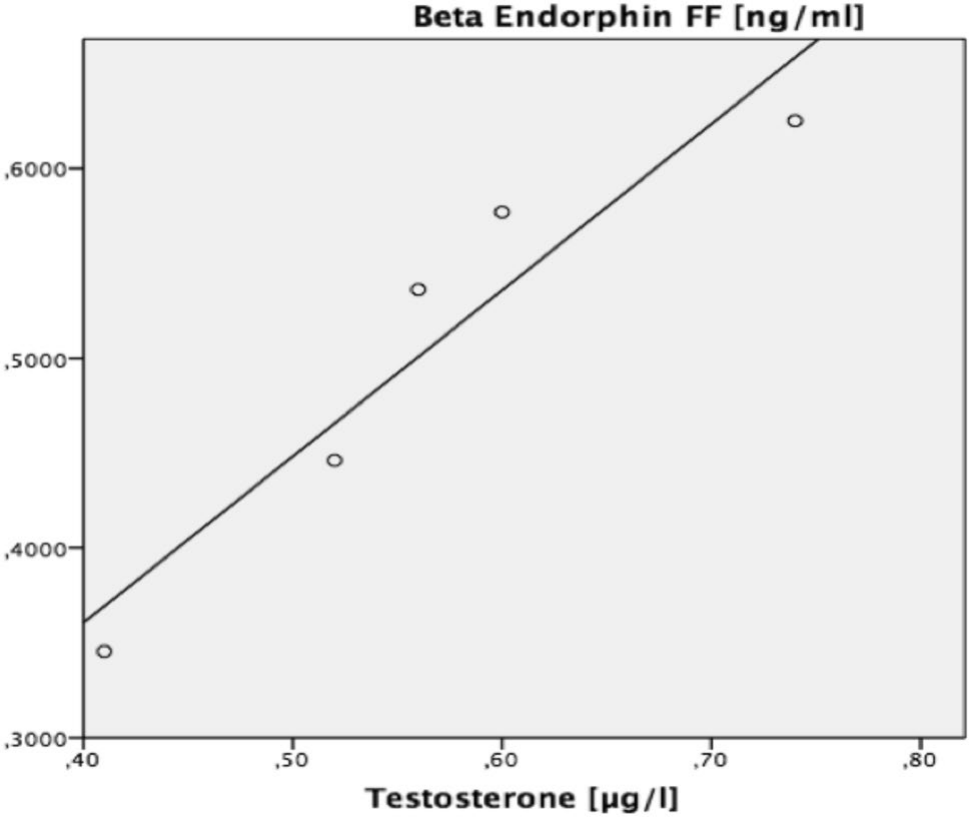
Regression analysis in women with biochemical hyperandrogenemia. Dependent variable: beta endorphin in FF, independent variable: testosterone

Based on previously published data that androgens regulate granulosa cell POMC expression [20], we evaluated women with PCOS and biochemical hyperandrogenemia (*n* = 5) and repeated the above described regression analysis, showing that T was the only highly predictive variable for beta endorphin levels in FF (*r*^2^ = 0.899, *p* = 0.014) (Fig. 2).

## Discussion

While the neuroendocrine regulation of follicle maturation has been well known for decades, elucidating the complex para- and autocrine mechanisms in the ovary has proven to be difficult. Over 30 years ago, Petraglia et al. [15] reported higher beta endorphin concentrations in FF than in serum and declining concentrations of the peptide in follicular fluid in the luteal phase. We have detected the beta endorphin precursor POMC in primary granulosa cells using immunofluorescence technique (unpublished data, Lunger F.), thus verifying a local production of endogenous opioids in the ovary. Furthermore, a basal POMC expression has been described in porcine granulosa cells [21]. Therefore, we expected higher concentrations of beta endorphin in FF than in serum in line with a local production. In fact, we even found lower beta endorphin concentrations in FF compared to serum. A possible explanation for this observation is the cycle phase in which the FF was collected, namely post-ovulation trigger on the day of oocyte retrieval, corresponding to the early luteal phase. In mouse models, beta endorphin concentration in FF significantly falls 10 h after hCG administration [22]. Hence, the timeframe in our study between ovulation induction and FF collection is sufficient to explain the lower concentrations in FF. Also, Petraglia et al. found higher beta endorphin concentrations in FF when collected in follicular phase. We did not find a correlation between FF and serum concentration of beta endorphin, thus supporting the concept of a local follicular regulation of beta endorphin expression, secretion and clearance.

Our findings are not consistent with those of Kialka et al., who reported higher beta endorphin concentrations in the serum of PCOS women compared to controls, independent of the BMI [23]. However, the authors used the NIH criteria for PCOS and included only women with biochemical hyperandrogenemia in the PCOS group, while we used the Rotterdam criteria. We did not have sufficient women in the PCOS group to perform extensive subgroup analysis based on PCOS phenotype. Moreover, we collected serum samples in the context of IVF and we do not know how the stimulation protocols may impact beta endorphin concentrations in serum.

The most intriguing finding of our study is the positive correlation between the proportion of mature metaphase II oocytes (MII) and beta endorphin content in FF. Beta endorphin was a strong independent predictor of retrieved MII oocytes even when controlling for age. Agirregoitia et al. have shown that G-Protein coupled (GPC) opioid receptors are expressed on human oocytes and that the localization of the µ-opioid receptor changes during oocyte maturation. While in germinal vesicle (GV) phase the receptor is mainly found peripherally (plasma membrane), a more homogenous distribution of the receptor in MII has been described, thus indicating an internalization during oocyte maturation [17]. After ligand binding (activation), GPC opioid receptors are desensitized and internalized [24]. As the µ-opioid receptor has been found to be mainly internalized in MII and the beta endorphin content of FF correlates with the number of retrieved MII oocytes, one could speculate that the activation (and sequential internalization) of µ-opioid receptors through beta endorphin binding promotes meiosis progression and therefore influences oocyte maturation.

The presence of sex steroids seems to be critical for beta endorphin’s influence on oocyte maturation. In bovine oocyte maturation, an inhibitory effect of beta endorphin has been demonstrated, but only in the absence of sex hormones [25]. This is consistent with the known effects of beta endorphin in the human pancreas and hypothalamus, where the presence of sex hormones is pivotal for stimulating insulin/glucagon release and inhibition of GnRH pulsatility, respectively [5, 26].

Our finding that testosterone levels positively correlated with the number of MII oocytes is consistent with the studies that have reported beneficial effects of androgens on follicular maturation and oocyte yield [27, 28].

In our experiment, the correlation between beta endorphin content in FF and testosterone was only significant in women with biochemical hyperandrogenemia, suggesting that 90% of variation of beta endorphin concentrations in FF was explained by testosterone levels. This may be analogous to the known physiology of androgens in both animal models as well as in the human prostate, where a critical androgen threshold needs to be reached before androgens can exert their regular actions in vitro [29].

Our study has several limitations. The sample size of PCOS women is relatively small. We pooled the FF of the first two follicles from each side to avoid dilution due to subsequent flushing. We purposely did not pool all the FF from larger and smaller follicles. Thus, we cannot make any statement regarding relationship between follicle size, oocyte maturity and beta endorphin content per follicle. We acknowledge that the follicular fluid content of beta endorphin may differ per follicle. Furthermore, we did not measure testosterone on retrieval day, but before starting the stimulation protocol. One study [30] has found a small increase of serum testosterone mid-cycle which may serve as an explanation for the high pre-ovulatory beta endorphin concentrations in FF as described by Petraglia et al.

In summary, to the best of our knowledge, we are the first to report a potential role of beta endorphin in oocyte maturation in humans. The lack of correlation between serum and follicular fluid levels supports the hypothesis of a local expression, secretion and clearance of the peptide. When testosterone levels are high, beta endorphin content in follicular fluid might be almost exclusively regulated by androgens. We do not know what other molecules are involved in regulating granulosa cell POMC expression and if these factors are secreted systemically, locally, or both. Future studies elucidating these regulating factors will improve our understanding of opioid physiology in the ovary.
